# Transfer RNA-derived small RNAs in the cancer transcriptome

**DOI:** 10.1007/s00424-016-1822-9

**Published:** 2016-04-20

**Authors:** Darrell Green, William D. Fraser, Tamas Dalmay

**Affiliations:** Norwich Medical School, University of East Anglia, Norwich Research Park, Norwich, NR4 7TJ UK; Department of Endocrinology, Norfolk and Norwich University Hospital, Norwich Research Park, Norwich, NR4 7UY UK; School of Biological Sciences, University of East Anglia, Norwich Research Park, Norwich, NR4 7TJ UK

**Keywords:** Non-coding RNA, tRNA, Small RNA, RNA silencing, Cancer

## Abstract

The cellular lifetime includes stages such as differentiation, proliferation, division, senescence and apoptosis. These stages are driven by a strictly ordered process of transcription dynamics. Molecular disruption to RNA polymerase assembly, chromatin remodelling and transcription factor binding through to RNA editing, splicing, post-transcriptional regulation and ribosome scanning can result in significant costs arising from genome instability. Cancer development is one example of when such disruption takes place. RNA silencing is a term used to describe the effects of post-transcriptional gene silencing mediated by a diverse set of small RNA molecules. Small RNAs are crucial for regulating gene expression and microguarding genome integrity. RNA silencing studies predominantly focus on small RNAs such as microRNAs, short-interfering RNAs and piwi-interacting RNAs. We describe an emerging renewal of interest in a ‘larger’ small RNA, the transfer RNA (tRNA). Precisely generated tRNA-derived small RNAs, named tRNA halves (tiRNAs) and tRNA fragments (tRFs), have been reported to be abundant with dysregulation associated with cancer. Transfection of tiRNAs inhibits protein translation by displacing eukaryotic initiation factors from messenger RNA (mRNA) and inaugurating stress granule formation. Knockdown of an overexpressed tRF inhibits cancer cell proliferation. Recovery of lacking tRFs prevents cancer metastasis. The dual oncogenic and tumour-suppressive role is typical of functional small RNAs. We review recent reports on tiRNA and tRF discovery and biogenesis, identification and analysis from next-generation sequencing data and a mechanistic animal study to demonstrate their physiological role in cancer biology. We propose tRNA-derived small RNA-mediated RNA silencing is an innate defence mechanism to prevent oncogenic translation. We expect that cancer cells are percipient to their ablated control of transcription and attempt to prevent loss of genome control through RNA silencing.

## Introduction

Transcription is a core step in the regulation of gene expression. It is of fundamental importance in maintaining organism function and integrity. The transcription process involves a number of active and intricate molecular interactions. The inherent transcription machinery and associated cofactors are dynamically recruited to their target DNA loci in order to produce messenger RNA (mRNA) transcripts, actively expressing a gene. Interactions between DNA-binding factors and chromatin play a key role in RNA polymerase assembly, initiation, and elongation [12]. The short lasting contact between transcription factors and DNA is superimposed by intermittent chromatin remodelling and binding events [12]. This cyclical and loci-selective nature not only is a central property of the transcription machinery but also has emerged as an important modulator of physiological processes such as differentiation, proliferation, and apoptosis.

Perturbation of these molecular synergies can result in significant fitness costs arising from genome instability. These costs may lead to accelerated ageing and disease. Neoplasia is a general term used to describe the effects of uncontrolled cellular division. These division effects may be corrected by immune responses or medical intervention. Malignant neoplasia, or cancer, is associated with abnormal, immature, and uncontrolled cell growth with a potential to spread to other parts of the body. Cancer typically evades immune responses and drastic curative measures are critical. Such measures include systemic chemotherapies and wide resection surgery. There are over two hundred cell types in the human body, most with their own distinctive pathways of homeostasis. Abhorrent cell division may arise from a variety of biological errors. Many cancer types are caused by somatic mutations to the genomic DNA molecule. Single nucleotide polymorphisms can cause DNA sequence variation. This may be beneficial in terms of organism or species evolution. It may also introduce deleterious effects. For instance, the open reading frame of transcription may be altered and protein production is disordered. Concerning the ~350 of our ~20,000 genes implicated in cancer development, DNA sequence variation may be lethal [31]. On a larger cytogenetic scale, chromosome structural variation is a normal part of human genome variation. Complex reshuffling of the genome may be harmful in the form of chromothripsis-related tumours and cancers formed as a result of fusion gene translocations [5, 28, 32].

The advent of DNA microarrays and next-generation sequencing, creating a post-genomic era of cancer genome projects, has been instrumental in delineating molecular subtypes of cancer. Many subtypes are associated with discrete biological infrastructures leading to varied disease progression and treatment responses [27]. The same technology used to decipher the molecular heterogeneity of cancer can be used to evaluate cancer transcriptome data, gaining greater biological insight [27]. Rather than focusing on and attempting to rectify the drivers of tumourigenesis, an alternative approach may be to refresh our understanding of cancer by studying and aiding innate molecular defence mechanisms. Defective coding from a transformed genome could be corrected by non-coding regulation.

## Small RNA biology

Small RNAs are a diverse set of functional, non-coding RNA molecules that are key regulators of gene expression through the process of gene silencing [2, 11]. Historically, ‘small RNA’ referred to any class of non-coding RNA 50–200 nucleotides (nt) in length. Over the last 15 years, the term is specifically used to describe ‘smaller’ small RNAs of 19–32 nt [25]. RNA polymerase II transcribes precursor molecules for most small RNAs such as microRNAs (miRNAs) and piwi-interacting RNAs (piRNAs) in a similar manner to that of protein coding genes. Some small RNAs are generated from structure-specific cleavages of other parent non-coding RNAs such as Y RNAs and tRNAs which are transcribed by RNA polymerase III [13, 24]. Small RNA research is proceeding at an astoundingly fast rate. There is ever increasing knowledge of the functions of the various classes of small RNA as well as the pathways they operate to alter gene regulation [11].

**Fig. 1:**
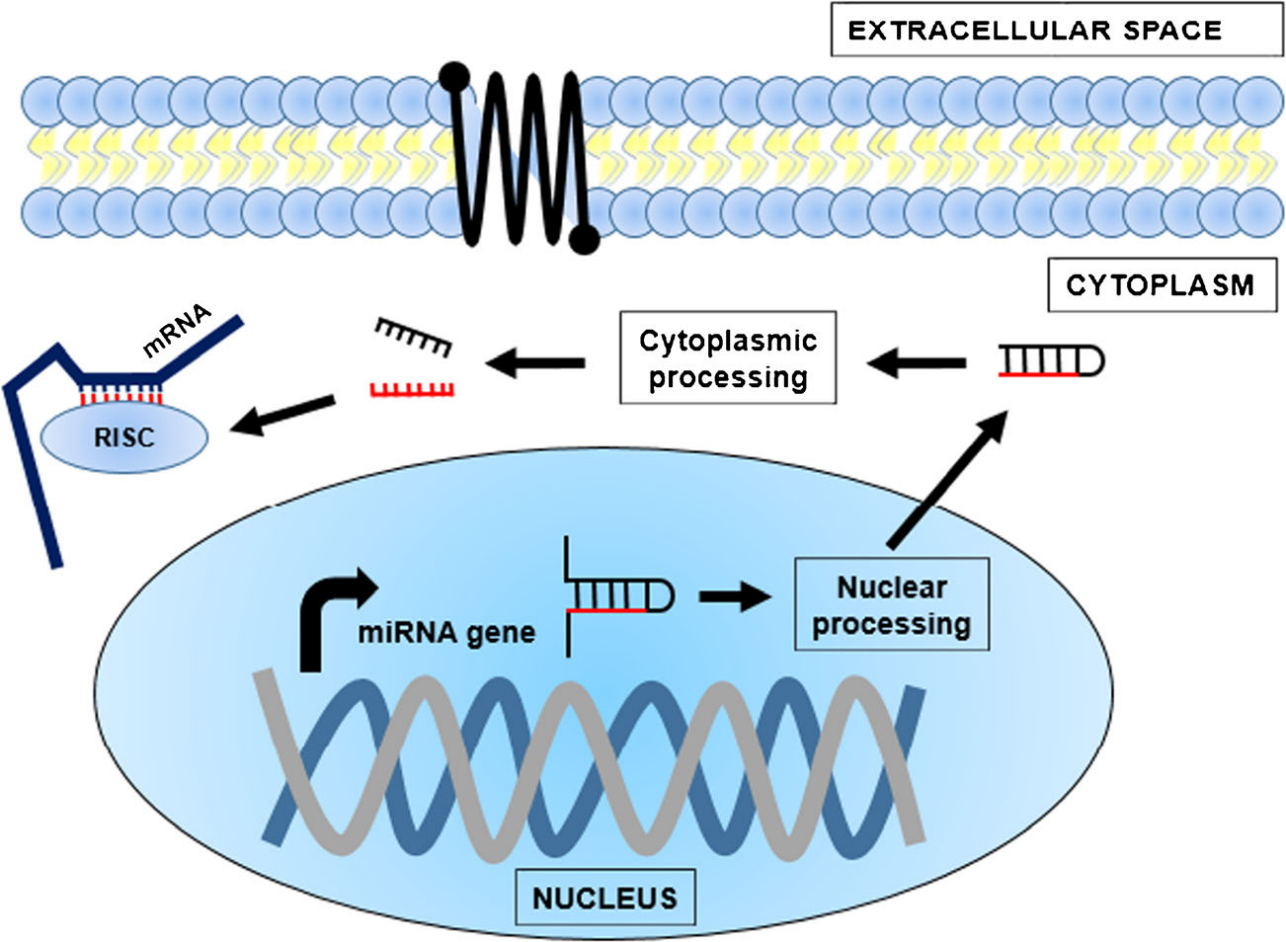
Biogenesis and mode of action of miRNAs in RNA silencing. A miRNA gene is expressed from the genome where it undergoes processing in the nucleus before exportation to the cytoplasm. It undergoes further processing to create a mature ~22 nt miRNA (*in red*). The miRNA binds to a multi-domain protein assembly forming a ribonucleoprotein complex known as the RNA-induced silencing complex (RISC). RISC uses the miRNA sequence to bind to complementary sequences in target mRNAs and physically obstruct translation. RISC also contains an Argonaute protein which is capable of cleaving the mRNA if there is perfect base pairing with the miRNA seed sequence. This type of post-transcriptional gene silencing takes place for up to two-thirds of genes in humans

There is great interest in small RNAs because of their role in modifying gene expression through RNA-mediated gene silencing mechanisms [9]. RNA silencing is a term used for small RNA-mediated post-transcriptional gene silencing [11]. The increasing variety of eukaryotic processes that small RNAs are found to be involved in extends to transgenerational inheritance and epigenetic memory [26]. The most prominent and well-studied class of eukaryotic small RNA is the miRNAs which are best known for silencing and fine-tuning the expression of mRNAs as part of the RNA-induced silencing complex (RISC) (Fig. 1). RISC uses the miRNA sequence to target complementary mRNA sequences in order to physically obstruct translation. Matching between the miRNA and mRNA can take place at the miRNA seed sequence (nucleotides 2–8), the non-seed sequence (nucleotides 8+), or a combination of both. miRNAs are evolutionarily conserved and may silence one to many hundreds of genes [2]. It is accepted that miRNAs do not act as ‘on/off’ switches but rather continual ‘fine-tuners’ of gene expression [11].

**Fig. 2:**
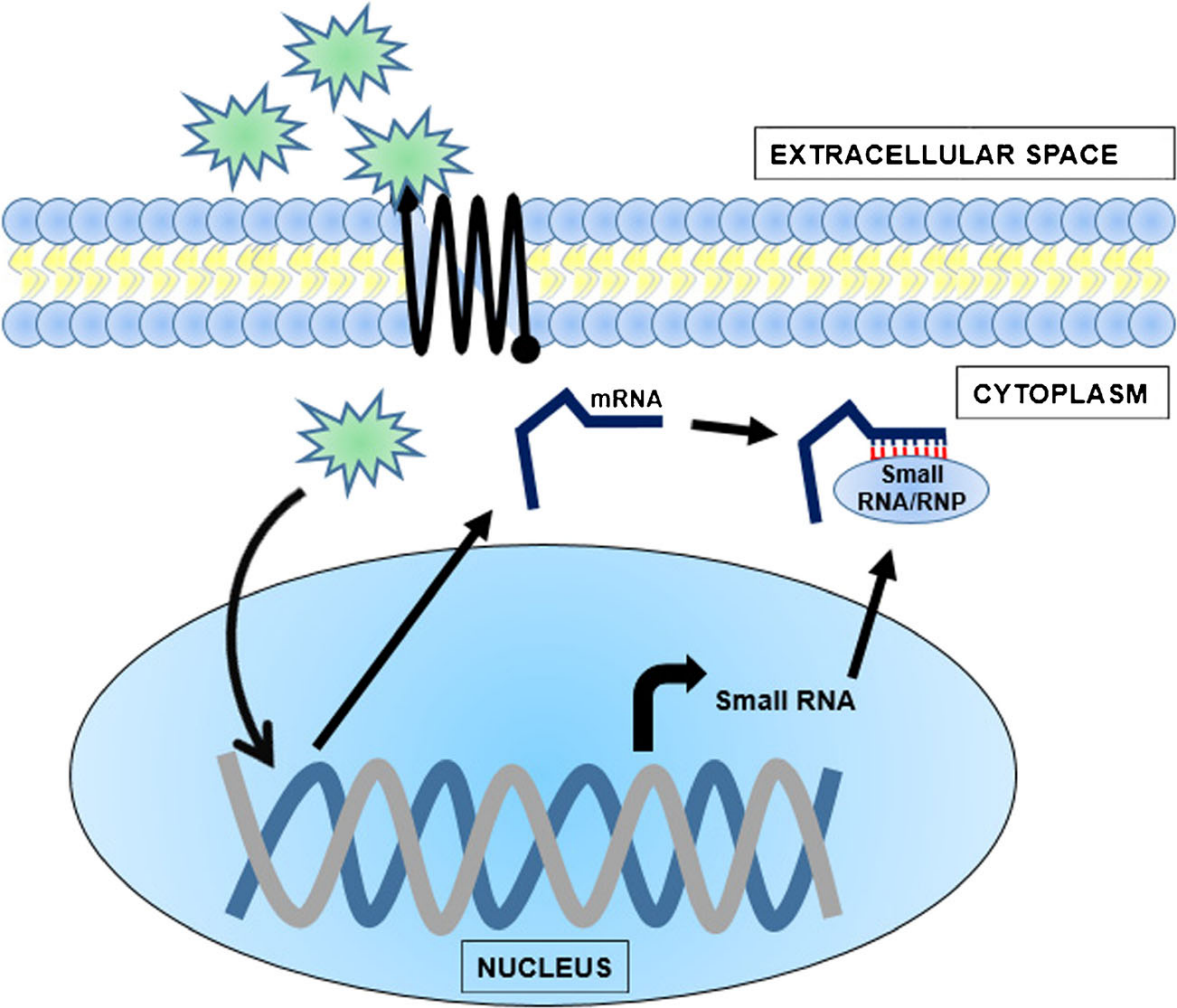
The basic premise of microguarding. External signals such as carcinogens or products of sexual reproduction (*in green*) may enter the cell through receptors and cause genome instability by triggering a fluctuation of abhorrent gene expression. In response, small RNAs (*in red*) are rapidly switched on to form a silencing ribonucleoprotein complex (RNP) to counteract this deleterious, exogenously induced transcription

All species have adapted to survive in the face of short-term variation in extrinsic and intrinsic stresses, such as nutrition, temperature, and reproduction. Bombardment from damaging exogenous factors such as carcinogens may overwhelm stress protection responses and accelerate cellular ageing. A recent hypothesis suggests that this kind of genetic inflammation could be minimised through the activity of miRNAs in a process known as microguarding [11]. Reports show that in organisms undergoing rapid change—for example during development or in response to periodic external perturbations such as sexual reproduction—miRNAs can switch from gene regulators to molecular guards [7, 11, 17]. In this latter role, miRNAs may buffer against abrupt fluctuations in mRNA transcription (Fig. 2). This can be important to prevent deleterious effects of variation of mRNA transcript abundance and minimising genetic instability leading to molecular-based disorders.

Due to their major role in modifying gene expression, miRNAs have been the focus of many cancer studies. Molecular approaches to profile genome-wide miRNA expression, i.e., library construction and next-generation sequencing, identified other types of small RNA which could not be assigned to miRNA loci during bioinformatics analysis. Many of the sequencing reads mapped to mRNAs, ribosomal RNAs and transfer RNAs (tRNAs). This data was initially dismissed as RNA degradation. On further analysis, the non-miRNA reads were realised to be abundant with recurring sequences, implicating the existence of novel small RNA classes. These ‘new’ small RNAs may play an important role in RNA silencing, microguarding and cancer.

## Discovery and role of tRNA-derived small RNAs

**Fig. 3:**
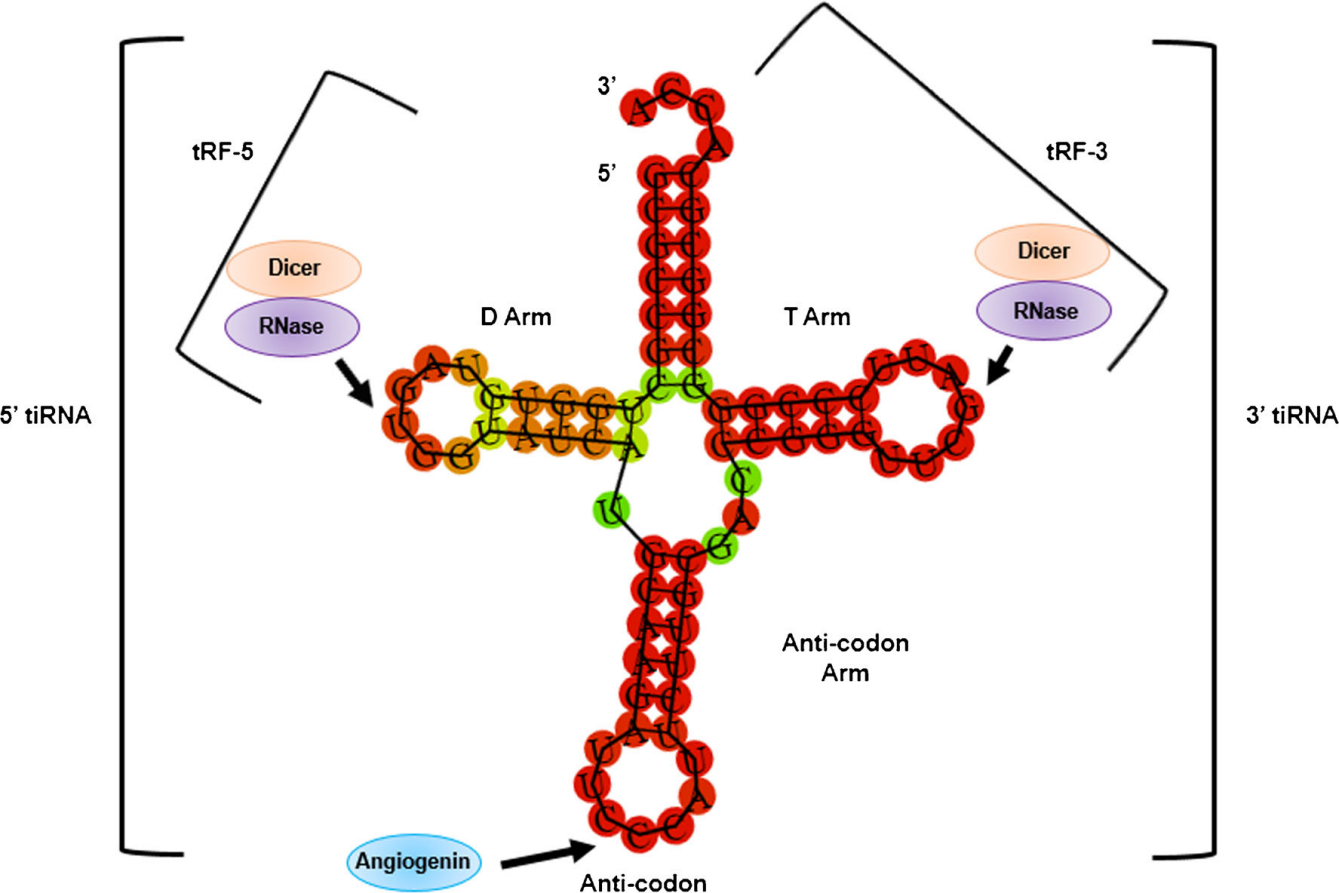
Schematic of tRNA-derived small RNA biogenesis from a mature tRNA^Gly^ molecule. The stress-activated ribonuclease Angiogenin cleaves the mature tRNA in the anticodon loop to produce two tRNA halves or tiRNAs. The production of tRF-3s is derived from a cleavage in the 3′ T arm loop (as the *arrow* indicates). The production of tRF-5s is derived from a cleavage in the 5′ D arm loop (as the *arrow* indicates). Conflicting reports show these cleavages to be Dicer-dependent and Dicer-independent. It is theorised that an RNase generates the majority of tRF-3s and tRF-5s instead of Dicer. In the nucleus, RNase Z produces tRF-1s from the trailer sequence of a precursor tRNA (not shown)

The best studied class of these new small RNAs are tRNA halves (tiRNAs). First described in *Escherichia coli* as a mechanism to antagonise bacteriophage infection, tiRNAs have been reported in numerous organisms [21, 33]. The stress-activated ribonuclease angiogenin cleaves mature tRNAs within anti-codon loops to produce 5′ and 3′ tiRNAs [16] (Fig. 3). Transfection of natural or synthetic 5′ tiRNAs inhibited protein translation in bone cancer cells by displacing eukaryotic initiation factors (eiFs) 4B, 4E and 4G from the m^7^G cap of mRNA [16]. These initiation factors are vital for resolving secondary structure conformations in the 5′-UTR of mRNAs during ribosome scanning [14]. Natural and synthetic 5′ tiRNAs have also been reported to induce the phospho-eiF2α-independent assembly of stress granules [16]. Stress granules are cytoplasmic RNA multimeric bodies that form under stress and are known to inhibit protein translation [1]. In most reported cases, formation of stress granules is associated with cell recovery, survival and cancer cell resistance to chemotherapeutics [1]. The finding that angiogenin contributes to stress-induced translational suppression suggests that tiRNAs help to reprogram protein translation during times of cellular stress [16, 36].

Distinct but related to tiRNAs are the less well studied tRNA fragments (tRFs). Libraries of 17–26 nt small RNAs were constructed from prostate adenocarcinoma cells prior to next-generation sequencing. A significant number of sequences were derived from the precise processing of 5′ and 3′ ends of mature or precursor tRNAs [20]. These differ to tiRNAs in that they are generated independent of angiogenin and are not cleaved within the anti-codon loop. These sequences were classified into tRF-5s, tRF-3s and those that map to the 3′ trailer fragment of precursor tRNAs (tRF-1 s) [20] (Fig. 3). To demonstrate the biological relevance of tRFs, the study investigated tRF-1001 which is derived from the 3′ end of a Ser-TGA tRNA precursor transcript that is not retained in the mature tRNA^Ser^ molecule [20]. Knockdown of tRF-1001 impaired normal cell proliferation with a distinct accumulation of cells in the G2 phase of the cell cycle [20]. Reduced proliferation arising from increased cell density or serum depletion was rescued by co-introduction of a synthetic 2′-*O*-methyl tRF-1001 oligonucleotide [20]. The tRF-1001 molecule was reported to be generated in the cytoplasm by tRNA 3′ endonuclease ElaC ribonuclease Z 2 (ELAC2), a known prostate cancer susceptibility protein, suggesting an oncogenic role for tRF-1001 [20].

Later, tRF-5s and tRF-3s were observed to behave similarly to miRNAs in human cell lines. The tRFs had associations with Argonaute proteins, and one tRF was shown to be produced by Dicer, the enzyme responsible for cytoplasmic miRNA processing [4, 15]. The latter study further demonstrated tRF-1s preferentially associated with Argonaute 3 and 4 [15]. It is known that miRNAs interact with all four Argonaute proteins [6]. The deliberate sorting of different types of small RNA, despite miRNAs and tRFs having almost identical properties, indicates an exclusive role for tRFs in cell biology.

## Meta-analysis of tRF biology

Since the discovery of tRFs, there have been several studies in a variety of model organisms. These studies conflict on the biogenesis of tRF-5s and tRFs-3s with some implicating Dicer and others reporting Dicer-independent pathways [22]. Several studies have shown that tRF-5s or tRF-3s can associate with Argonaute proteins and perform RNA silencing [3, 23, 35]. Other studies argue that tRF-5s cannot perform RNA silencing, instead functioning in a similar manner to that of 5′ tiRNAs in protein translation inhibition [30].

To address the contentions on tRF biogenesis and function, Kumar et al. took advantage of the deposited small RNA data to perform a meta-analysis of tRFs and provide an inference to their biology [18]. The investigation looked at 50 small RNA datasets and concluded that tRFs are precisely generated fragments and are not produced by the miRNA biogenesis pathway [18]. Though some tRFs were discovered to be produced by Dicer, it is more likely that other RNase enzymes (such as RNase P and RNase Z) are responsible for the majority of tRF generation [18]. Human photoactivatable-ribonucleoside-enhanced crosslinking and immunoprecipitation (PAR-CLIP) data showed a preference for tRF-5s and tRF-3s to associate with Argonaute 1, 3 and 4 rather than Argonaute 2 [18]. Analysis of positional T to C mutational frequency indicated that tRFs associate to Argonaute proteins in a similar fashion to that of miRNAs. The reverse complements of canonical seed sequences matched crosslink-centred regions, suggesting that tRF-5s and tRF-3s interact with thousands of different RNAs in the cell [18]. The meta-analysis concludes that tRFs are an abundant class of small RNA whose biogenesis is distinct from miRNAs, but they have similar properties and may play a major role in RNA silencing [18].

Despite the fact that tRFs are more evolutionarily conserved than miRNAs, tRFs are present in similar abundance to miRNAs and display Argonaute sorting in humans, there was not a global repository for tRF sequences. This lack of consensus resulted in multiple labs working on the same tRF without realising, for example, cand45 in Cole et al. [4] is the same molecule as tRF-1001 in Lee et al. [20]. Moreover, a tRF may be misannotated as a miRNA [29]. To address this issue, Kumar et al. created a database of tRFs known as tRFdb available at www.genome.bioch.virginia.edu/trfdb/ [19].

## tRFs prevent cancer metastasis

It was proposed that tiRNAs and tRFs could have roles in cancer progression, similar to the role of miRNAs. As hypoxia is a major stress encountered by cancer cells, tRNA-derived small RNAs induced under hypoxic conditions may act to curb metastatic progression [10]. This theory fits in line with microguarding concepts where cancer cells may be intuitive to their ablated control of transcription and attempt to prevent loss of genome control through RNA silencing [11]. Vice versa, it may be a mechanism for a cancer cell to produce stress granules, inhibit protein translation, and reactivate when the microenvironment is more suitable to tumour formation and growth.

A group of tRFs were identified by small RNA sequencing that were upregulated under hypoxia in breast cancer cells as well as non-transformed mammary epithelial cells [10]. Highly metastatic breast cancer cells did not display induction of the same tRFs under hypoxia, suggesting a role specific to cancer progression [10]. The study identified a common sequence motif present in the hypoxia-induced fragments, suggesting that they interact with a common trans-factor. Using tRF^Glu^ as ‘bait’, the RNA-binding protein Y box binding protein 1 (YBX1) was immunoprecipitated and identified as the trans-factor [10]. The mRNA stabilising activity of YBX1 is suppressed by tRFs.

**Fig. 4:**
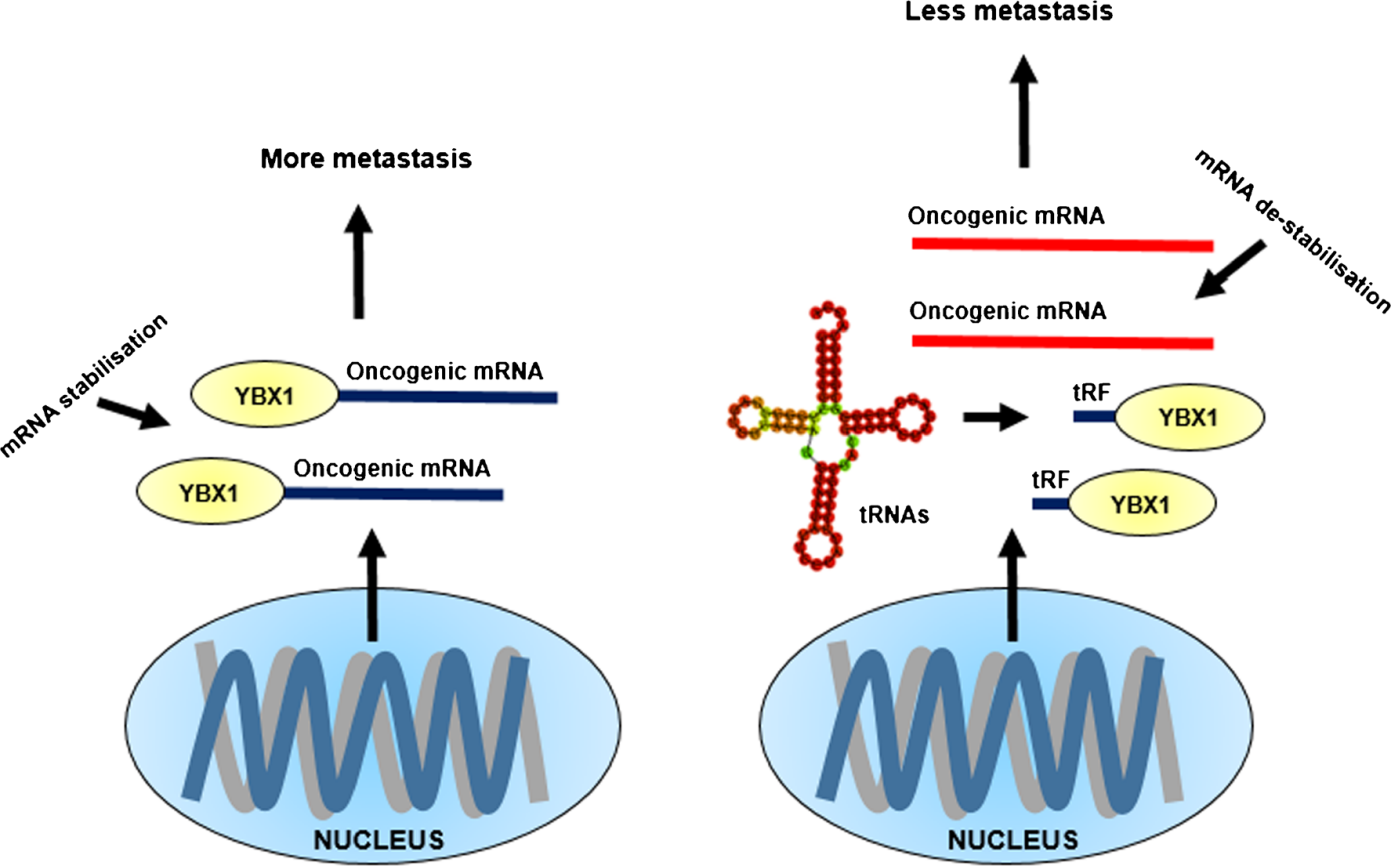
Oncogenic genes are expressed by the cancer cell which promote its survival and metastasis. YBX1 is an RNA-binding protein which stabilises the oncogenic transcripts for translation, resulting in more metastasis (*left-hand side*). The cancer cell increases production of tRNA-derived small RNAs, which compete for YBX1 binding. Oncogenic transcripts are destabilised (in *red*), and translation is diminished, resulting in less metastasis (*right-hand side*). This innate system is open to manipulation through the use of RNA mimics and inhibitors

YBX1 is a malleable RNA-binding protein with a variety of networking partners. It is involved in key cellular pathways and inactivation leads to embryonic lethality [34]. YBX1 is also overexpressed in multiple cancer types. By combining molecular, biochemical and computational approaches, it was reported that tRFs bind to YBX1 and displace a number of oncogenic transcripts from YBX1 [10]. Antagonising YBX1 activity interferes with its role in stabilising the expression of oncogenic mRNAs (Fig. 4). The displacement of these oncogenic transcripts by tRFs suppresses their stability and expression, therefore suppressing metastatic progression [10].

To demonstrate the physiological significance of the hypoxia-induced tRF-YBX1 pathway, the study further used a reporter to drive the expression of luciferase under a hypoxia response promoter in mice xenografted with human breast cancer. A lentiviral system with a luciferase reporter fused to 3′-UTRs of cluster of differentiation 97 (CD97) and TIMP metallopeptidase inhibitor 3 (TIMP3), two important tRF-YBX1 targets, was used to show lower luciferase activity in YBX1 knockdowns post-injection [10]. The lack of luciferase activity from reporters fused with CD97 and TIMP3 is direct evidence of tRF-mediated displacement of oncogenic transcripts from YBX1 [10].

## Summary and conclusions

Transfer RNA-derived small RNAs are amongst a diverse set of small RNA molecules present in bacteria to humans. These tiRNAs and tRFs can be freely generated from the transcriptome or produced by stress-induced cleavages. Non-stress-induced tRNA-derived small RNAs are thought to arise from ribonucleolytic processing of tRNAs by Dicer and RNase Z [4, 20]. The production of stress-induced tRNA-derived small RNAs has been shown to occur via the action of other ribonucleases such as angiogenin [8]. Multiple classes of tiRNAs and tRFs have been identified in various cell types and organisms. These classes are defined by the position of the tRNA cleavage site that gives rise to the small RNAs. These include 5′ and 3′ tRNA halves that are cleaved within the anti-codon loop [20]. These classes also include tRF-5s, tRF-3s and tRF-1s which are cleaved within the tRNA arm loops or trailer sequence [18]. Stress-induced tiRNAs have been reported to mediate a stress response which results in stress granule assembly and inhibition of protein synthesis. Additionally, tRFs can impact on a number of cellular functions including proliferation and mediating RNA inactivation through Argonaute co-operation.

We propose that tRNA-derived small RNAs are an innate tumour suppressive mechanism which is open to molecular manipulation through the use of RNA mimicry and antagonism. Some reports conflict with this idea where some tRNA-derived small RNAs promote cancer. This dual role as oncogenic and tumour suppressive is typical of functional small RNAs. It may also be evidence of the tumour evolutionary arms race for metastatic clonal selection. We previously suggested that miRNAs can buffer against potential oncogenic transcription [11]. Research is needed to identify other small RNAs or mechanisms that might have a more significant role in microguarding [11]. The costs of defective microguarding were expected to result in accelerated ageing and increased disposition to disease. In this review, we shift focus away from miRNAs and discuss the role of tRNA-derived small RNAs in preventing protein translation and in displacement of YBX1. This added component to the microguarding phenomena of small RNA-induced prevention of oncogenic transcription and translation is a further exciting avenue for future research.
